# Decorin–induced, preeclampsia-associated microRNA-512-3p restrains extravillous trophoblast functions by targeting USF2/PPP3R1 axis

**DOI:** 10.3389/fcell.2022.1014672

**Published:** 2022-10-10

**Authors:** Chidambra D. Halari, Pinki Nandi, Jasmin Sidhu, Maria Sbirnac, Michael Zheng, Peeyush K. Lala

**Affiliations:** ^1^ Department of Anatomy and Cell Biology, Schulich School of Medicine and Dentistry, University of Western Ontario, London, ON, Canada; ^2^ Children’s Health Research Institute, Schulich School of Medicine and Dentistry, University of Western Ontario, London, ON, Canada

**Keywords:** decorin, preeclampsia, microRNA, extravillous trophoblast, USF2, *PPP3R1*

## Abstract

Decorin (DCN) is a leucine-rich proteoglycan produced by chorionic villus mesenchymal cells anddecidual cells during human pregnancy. Studies from our laboratory demonstrated that decidua-derived DCN restrains multiple trophoblast functions including proliferation, migration, invasion andendovascular differentiation, mediated by DCN-binding to multiple tyrosine kinase receptors; expressed by the trophoblast. Furthermore, DCN was shown to be selectively over-produced by thedecidua in preeclampsia (PE) subjects and elevated in the second trimester maternal plasma in PE, before the appearance of clinical signs, presenting as a predictive biomarker for PE. Micro (mi)RNAs are single-stranded non-coding RNAs (17–25 nucleotides) that typically downregulate target genes by repressing translation or facilitating degradation of mRNAs. The human; placenta expresses many miRNAs, some of which are exclusively expressed by the trophoblast. Many; of these miRNAs are dysregulated in PE-associated placentas and some appear in the maternal blood as PE biomarkers. However, little is known about their contribution to the pathogenesis of PE, a multi-factorial disease associated with a hypo-invasive placenta. The objective of the present study was to examine whether exposure of extravillous trophoblast (EVT) to DCN affects expression of specific miRNAs, and to test the role of these miRNAs in altering EVT functions. We identified miR-512-3p, as one of the DCN-induced miRNAs, also upregulated in PE placentas. It was shown to be elevated in ectopic DCN-over-expressing or exogenous DCN-treated first trimester human trophoblast cell line HTR-8/SVneo. Use of miRNA-mimics and inhibitors revealed that miR-512-3p compromised trophoblast migration, invasion and VEGF-dependent endovascular differentiation. Finally, Protein Phosphatase 3 Regulatory Subunit B, Alpha (PPP3R1), a known target of miR-512-3p, was paradoxically elevated in miR-512-3p-overexpressing trophoblast and PE-associated placentas. Using Enrichr, a tool that consists of both a validated user-submitted gene list and a search engine for transcription factors, we found that PPP3R1 elevation resulted from the miRNA binding to and targeting Upstream Transcription Factor 2 (USF2) which targeted PPP3R1. These findings reveal a novel aspect of pathogenesis of PE and biomarker potentials of this miRNA in PE.

## Introduction

Preeclampsia (PE) is a severe pregnancy-specific disorder that affects around 5% of pregnancies worldwide. It is characterized by new onset hypertension and (typically) proteinuria after 20 weeks of gestation (Gao et al., 2018). If left untreated, PE may lead to multisystem organ damage such as renal failure, pancreatitis and hemolytic anemia (Sheikh et al., 2016). PE is one of the leading causes of maternal and perinatal morbidity and mortality (Duley, 2009). It has long been recognized that the pathogenesis of PE lies within the placenta because removal of the placenta eradicates the clinical manifestations of PE (Zhu et al., 2009; Chen and Wang, 2013).

During normal placental development, trophoblast stem cells contained within the cytotrophoblast (CTB) layer of the chorionic villi differentiate into two subpopulations: syncytiotrophoblast (STB) and extravillous trophoblast (EVT). STB arises by cell fusion and EVT as migratory cell columns. EVT cells proliferate at the villus base and invade uterine decidua and spiral arteries (Knöfler and Pollheimer, 2013). Some of the EVT cells undergo endothelial-like (endovascular) differentiation to invade and remodel distal segments of the arteries into low-resistance tubes that allow steady flow of maternal blood for fetal nourishment (Kaufmann et al., 2003; Cartwright et al., 2010). While both EVT and STB differentiation pathways are dysregulated in PE, poor EVT invasion is thought to be the root cause of the pathological manifestation such as defective uterine arterial remodeling, resulting in a hypo-perfused placenta (Kaufmann et al., 2003; Lala and Chakraborty, 2003; Burton et al., 2009; Cartwright et al., 2010). A hypoxic placenta releases toxic lipid peroxides and inflammatory chemokines into the maternal circulation, leading to vascular damage in multiple maternal organs (Roberts and Lain, 2002). Currently, there is no known cure for PE except delivering the fetus and the placenta (Backes et al., 2011). However, premature delivery increases perinatal morbidity and mortality rates (Backes et al., 2011). As such, further research to understand the mechanisms underlying PE and to identify the novel biomarkers for its early detection is urgently needed. Decorin (DCN), a leucine rich-proteoglycan produced by various mesenchymal cells including decidual stromal cells, is overexpressed by the PE-associated decidua, as shown by *in situ* hybridization for mRNA and immuno-localization of the protein (Siddiqui et al., 2016). Additionally, plasma DCN levels in second trimester patients are elevated in PE compared to control (non-PE) patients matched for body-mass index (Siddiqui et al., 2016). DCN controls EVT cell proliferation, migration, invasion (Xu et al., 2002; Iacob et al., 2008) and endovascular differentiation (Lala et al., 2012), events needed for uterine spiral artery remodeling (Kaufmann et al., 2003; Lala and Nandi, 2016). Furthermore, DCN is a key regulator of human trophoblast stem cell self-renewal and differentiation (Nandi et al., 2018) and plays an autocrine role in decidual cell differentiation from human endometrial stromal cells (HESC) (Halari et al., 2020). These findings reveal that a balanced DCN production by the decidua is essential for a healthy pregnancy. Decreased DCN production may result in decidual maturation defects, whereas increased levels may result in compromised trophoblast functions associated with PE.

MicroRNAs (miRNAs) are single-stranded non-coding RNAs (17–25 nucleotides) that typically bind to the 3′ untranslated region of target mRNAs to block translation and facilitate degradation (Dexheimer and Cochella, 2020). The human placenta expresses many miRNAs, some of which are exclusively expressed by trophoblasts and not by other normal human tissues (Baek et al., 2008). These placenta-specific miRNAs are clustered in three groups: chromosome 14 miRNA cluster (C14MC), chromosome 19 miRNA cluster (C19MC), and miR-371–373 cluster (Miura et al., 2010; Morales-Prieto et al., 2012). Some of them have been detected in maternal circulation throughout gestation with a significant decline after delivery (Miura et al., 2010; Kotlabova et al., 2011). Many of the miRNAs are dysregulated in PE-associated placentas (Ishibashi et al., 2012; Hong et al., 2014; Xu et al., 2021) and some may appear in the maternal blood as PE biomarkers (Munaut et al., 2016; Jelena et al., 2020; Xu et al., 2021). MiR-512-3p is part of the C19MC (Morales-Prieto and Markert, 2011) and multiple studies have reported upregulation of miR-512-3p in PE patients (Wang et al., 2012; Martinez-Fierro et al., 2018; Martinez-Fierro and Garza-Veloz, 2021). A recent study showed elevated level of this miR-512-3p at 20 weeks of gestation in the serum of women who later developed severe PE (Martinez-Fierro and Garza-Veloz, 2021). Whether this miRNA contributes to the pathology in PE has never been investigated. The objective of the present study was to determine whether exposure of EVTs to DCN affects expression of specific miRNAs, and to test the role of these miRNAs in altering EVT functions. We identified miR-512-3p, as one of the DCN-induced miRNAs, also upregulated in PE placentas. It was shown to be elevated in ectopic DCN-over-expressing or exogenous DCN-treated first trimester human trophoblast cell line HTR-8/SVneo. Use of miRNA-mimics and inhibitors revealed that miR-512-3p compromised trophoblast migration, invasion and VEGF-dependent endovascular differentiation. Surprisingly, *PPP3R1*, a known target of miR-512-3p, was paradoxically elevated in miR-512-3p overexpressing trophoblast and PE-associated placentas. Using Enrichr, a tool that consists of both a validated user-submitted gene list and a search engine for transcription factors, we found that *PPP3R1* elevation resulted from the miRNA binding to and targeting a transcription factor USF2 which targeted *PPP3R1*. These findings suggest that induction of this miRNA may be one mechanism for the pathogenesis of PE by DCN overproduction by the decidua.

## Materials and methods

### Cell line and culture

HTR-8/SVneo cells, originally produced by immortalization of EVTs derived from explant outgrowths (Graham et al., 1993), and commonly used as a model of invasive EVTs, were maintained in RPMI- 1640 supplemented with 10% fetal bovine serum (FBS), 100 units/ml penicillin, and 100 μM streptomycin. Cells were passaged *via* light trypsinization prior to reaching confluency and were maintained at 37°C in an atmosphere consisting of 5% CO_2_ for no more than twenty sequential passages.

### Placenta sample collection

Flash-frozen placenta samples collected from normotensive and preeclampsia were obtained from the Research Centre for Women’s and Children’s Health Biobank (RCWIH, Mount Sinai Hospital, Toronto, ON, Canada, http://biobank.lunenfeld.ca). All samples were collected from caesarean section deliveries with informed consent and were approved by the Mount Sinai Hospital and University of Western Ontario research ethics boards. Jeyarajah et al., 2019, have reported the details of these subjects including the gestational age of the pregnancies and parameters used to define preeclampsia.

### Transfection with microRNA-mimics and inhibitors

Prior to transfection, HTR-8/SVneo cells were plated in a 12-well plate and grown to 70%–80% confluency. Following the removal of culture media, the cells were incubated with 800 μl/well Opti-MEM media (supplemented with 7.5% FBS) for 1 h. Cells were then treated with Lipofectamine 2000 RNAiMax (Invitrogen, 2.5 μl Lipofectamine/100 μl of Opti-MEM media) and oligonucleotides (2 μg/well) and incubated for 4 h. Subsequently, cells were washed two times with RPMI-1640 complete media and incubated overnight. Successful transfection was verified by determining miRNA levels in cells 24 h after exposure to lipofectamine. MiRNA overexpression and knockdown were achieved *via* transfection with miRIDIAN miR-512-3p mimic (Dharmacon, cat no. C-300769-03-0005) and miR-512-3p Inhibitor (Dharmacon, Cat No. IH-300769-05-0005). Controls for overexpression and knockdown experiments consisted of transfection with miRIDIAN™ Dharmacon mimic (Dharmacon, Cat No. CN-001000-01-05) and inhibitor (Dharmacon, Cat No. IN-001005-01-05), respectively. The sequences of these control oligonucleotides do not target any known mammalian transcript.

### SiRNA-mediated *PPP3R1* knockdown

Transient transfection of *PPP3R1* siRNA oligomers (Thermofisher, cat no. AM16708) and negative control (Thermofisher, cat no AM4641) was carried out using lipofectamine 2000 RNAiMax (Invitrogen, Thermo Fisher). All plasmid transfections (1 μg) were carried out similar to the protocol described above in miRNA transfection. Successful transfection was verified by determining *PPP3R1* mRNA levels in cells 24 h after transfection using quantitative reverse transcriptase polymerase chain reaction (qRT-PCR). Experiments with *PPP3R1* knockdown cells were conducted within 3 days of transfection.

### MicroRNA extraction from HTR-8/SVneo cells

MiRNA was extracted from HTR-8/SVneo cells using the miRNeasy Kit (Qiagen) according to the manufacturer’s instructions (Qiagen, 2020). Briefly, HTR-8/SVneo cells were lysed using 700 µl of TRIzol (Thermofisher) and collected in 1.5 ml Eppendorf tubes. The resulting lysate was treated with 140 µl of chloroform, agitated for 15 s, and centrifuged at 12,000 rpm for 15 min (4°C). The upper aqueous phase was then transferred to a new collection tube and mixed in a 1:1 ratio with 70% ethanol solution. The resulting mixture was then put through the RNeasy minielute spin column (Qiagen, Cat No. 1026497) in increments of 700 µl and centrifuged at 10,000 rpm for 1 min (25°C). Flowthrough was collected, mixed with 0.65 volume of 100% ethanol, and passed through the miRNeasy column at 10,000 rpm for 1 min (25°C) (Qiagen, Cat No. 74104) to isolate miRNA. Once done, the resulting flowthrough was discarded and the isolated miRNA was washed in two steps: 500 μl of RPE buffer (Qiagen) was added, the column was centrifuged at 10,000 rpm for 1 min (25°C) and flow through was discarded; 500 μl of 80% ethanol was added, the column was centrifuged at 10,000 rpm for 1 min (25°C), and flowthrough was discarded. The column was then centrifuged at 10,000 rpm for 5 min (25°C) to dry. Lastly, the column was transferred to a new 1.5 ml Eppendorf collection tube, 12 μl of RNase-free water (Qiagen) was added, and the new tube was centrifuged at 10,000 rpm for 2 min at 25°C. Following purification, the sample concentration and 260/230-absorbance purity ratio were quantified using the Epoch/Take Multi-Volume Spectrophotometer System (BioTek Instruments, Inc.).

### MicroRNA extraction from placental tissue samples

MiRNA was extracted from placental tissue samples using the miRNeasy Kit (Qiagen) according to manufacturer’s instructions (Qiagen). Briefly, a small amount of flash-frozen tissue was placed in a 15 ml collection tube with 700 µl of TRIzol (Invitrogen Life Technologies) and homogenized for 40 s using the Omni TH Tissue Homogenizer (Omni International, Inc.). The tube was then left to sit at room temperature for 5 min to promote nucleoprotein complex dissociation. After 5 min, 140 µl chloroform was added to the tube and the isolation proceeded with the same protocol as the HTR-8/SVneo cellular miRNA extraction mentioned above.

### cDNA synthesis from microRNA

Purified miRNA was utilized for cDNA synthesis using the qScript miRNA cDNA Synthesis Kit (Quantabio) according to manufacturer’s instructions (Quantabio, 2020). Briefly, 2 µl of Poly(A) Tailing Buffer (5X), 1 µl of Poly(A) Polymerase, and 7 µl of 500 ng/μl purified miRNA solution were added to a PCR tube to give a final volume of 10 µl. The resulting mixture was then incubated in the C1000 thermal cycler (Bio-Rad) at 37°C for 60 min followed by incubation at 70°C for 5 min. Once finished incubating, 9 µl of miRNA cDNA Reaction Mix and 1 µl of qScript Reverse Transcriptase were added to the PCR tube to give a final volume of 20 µl. The resulting mixture was then placed back in the Thermal Cycler at 42°C for 20 min, followed by incubation at 85°C for 5 min. The resulting cDNA was stored at −20°C prior to qRT-PCR analyses.

### Reverse transcription-quantitative polymerase chain reaction

qRT-PCR was performed using the qScript miRNA cDNA Synthesis Kit (Quanta bio, Cat No. 95107-025) according to manufacturer’s instructions. Briefly, 2 μl of 500 ng/μl cDNA sample (diluted 1:10) was added to PCR tubes containing 18 μl of master mix (10 µl PerfeCTa SYBR Green SuperMix, 0.4 µl Custom miRNA Assay Primer (2 µM), 0.4 µl PerfeCTa Universal PCR Primer (10 µM), 7.2 µl Nuclease-Free Water). Samples were then run through the Rotor-Gene 3000 (Corbett Research) thermal cycler using custom designed primers (Tables 1, 2). Cycling conditions involved initial holding step (95°C for 13 min), followed by 45 cycles of a two-step PCR (95°C for 15 s and 60°C for 60 s) and a dissociation phase. Fold-changes in miRNA and mRNA expression in treated samples compared to control samples were calculated using the 2^−ΔΔCt^ method. RNU6 was used as a reference miRNA and the geometric mean of GAPDH and 18S rRNA (RNA18SN1) as reference RNA for HTR-8/SVneo and placental tissue samples.

**TABLE 1:** mRNA primer sequences used for qRT-PCR.

Gene	Forward primer	Reverse primer
*PPP3R1*	GAG​GGC​GTC​TCT​CAG​TTC​AG	GCT​GGA​CGT​CTT​GAG​CAG​AT
*USF2*	AAT​GGA​GGA​CAG​ACA​GGA​ACA​C	CTC​CTT​TAC​TCG​CTC​CCG​TC
*GAPDH*	AAT​GGG​CAG​CCG​TTA​GGA​AA	GCG​CCC​AAT​ACG​ACC​AAA​TC
*RNA18SN1*	GCA​ATT​ATT​CCC​CAT​GAA​CG	GGC​CTC​ACT​AAA​CCA​TCC​AA

Primers for quantitative real-time polymerase chain reaction (qRT-PCR): PPP3R1, Protein Phosphatase 3 Regulatory Subunit B, alpha; USF2, Upstream Transcription Factor 2; GAPDH, glyceraldehyde 3-phosphate dehydrogenase; RNA18SN1, 18S ribosomal N1.

**TABLE 2:** miRNA primer sequences used for qRT-PCR.

MiRNA	Forward primer
miR-512-3p	5′GTG​CTG​TCA​TAG​CTG​AGG​TCA​A3′
miR-512-5p	5′CTC​AGC​CTT​GAG​GGC​ACT​TT3′
miR-195-3p	5′GGC​TGT​GCT​GCT​CCA​AAA3′
miR-195-5p	5′GCT​AGC​AGC​ACA​GAA​ATA​TTG​G3′
miR-18b-3p	5′GCC​CTA​AAT​GCC​CCT​TCT​AAA3′
miR-18b-5p	5′GGT​GCA​TCT​AGT​GCA​GTT​AGA​AAA3′
miR-363-3p	5′CAA​TTG​CAC​GGT​ATC​CAT​CTG3′
miR-363-5p	5′GGG​TGG​ATC​ACG​ATG​CAA​T3′
miR-374a-3p	5′CGC​GCT​TAT​CAG​ATT​GTA​TTG​T3′
miR-374c-5p	5′CAT​AAT​ACA​ACC​TGC​TAA​GTG​CTA​AAA3′
miR-155-3p	5′GCT​CCT​ACA​TAT​TAG​CAT​TAA​CAA​AAA3′
miR-155-5p	5′TGC​TAA​TCG​TGA​TAG​GGG​TAA​A3′
let-7c-5p	5′-CCG​AGC​TGA​GGT​AGT​AGG​TTG​TAT​G-3′
U6	5′GCA​AAT​TCG​TGA​AGC​GTT​CC3′

### Wound healing assay

To measure migration, we used wound-healing assay. Cells were treated with mitomycin C (500 ng/ml, Sigma, cat: M4287) for 1 h to block cell proliferation, scratched multiple times (eight linear scratches each, vertically and horizontally) in the absence or presence of exogenous DCN (250 nM, a concentration previously shown to have the highest anti-migratory effect, Sigma, cat: D8428) for 24 h. The wound area was recorded using light microscopy (Leica Microsystems) at 0 and 24 h. Migration was recorded as the percentage wound closure at 24 h. To calculate the area of the wound, images were imported into ImageJ (version 1.5.3), where cell frontiers bordering the wound were traced. The percentage of wound closure was determined using the following equation: [(A0−A24)/A0] × 100%, where A0 represents the initial area of the wound at 0 h and A24 represents the area of the wound after incubating for 24 h.

### Transwell migration and invasion assays

A transwell migration assay was performed using a 24 well plate and transwell inserts (Corning, CLS3464) containing microporous (8 μm pores) membranes. 40,000 cells were resuspended in serum free media, placed on top of each transwell, and allowed to migrate through the microporous (8 μm pore) membrane for 24 h towards the complete FBS-containing media. After 24 h, non-migratory cells on top of the membrane were removed using a cotton bud and membranes were stained with hematoxylin and eosin. Once dried, membranes were imaged at ×40 total magnification using the Leica Inverted Light Microscope and cells were counted using ImageJ software. For the invasion assay, transwells were coated with a thin layer of matrigel (BD Biosciences, 400 μg/ml diluted in serum free RPMI-1640 medium) for 4 h before performing the assay same as above for 48 h.

### Spheroid invasion assay

This assay allows one to quantify the invasion of HTR-8/SVneo cells into Matrigel at various time points (Siddiqui et al., 2016). Spheroids were formed using 24-well AggreWell 800 plates (Stemcell Technologies) containing 800 μm microwells according to manufacturer’s protocol (Stemcell Technologies, 2017). Briefly, AggreWell 800 plate wells were pre-treated with 500 μl of Anti- Adherence Rinsing Solution (Stemcell Technologies, Cat No. 07010) and then the plate was centrifuged at 1,300 g for 5 min. Following microscope verification that no bubbles remained, the Anti-Adherence Rinsing Solution was aspirated from the wells. Each well was then rinsed with 2 ml of basal RPMI-1640 media prior to addition of 1 ml of complete RPMI-1640 media. Next, 900,000 HTR-8/SVneo cells suspended in 1 ml complete media were plated per well and the AggreWell plate was centrifuged at 100 g for 3 min. This resulted in approximately 3,000 HTR-8/SVneo cellscaptured in each microwell. The plate was then incubated at 37°C and 5% CO_2_ for 24 h. After 24 h, spheroids were collected using a P1000 pipette and passed through a 37 μm reversible strainer into a 15 ml conical tube. Next, 1 ml of basal RPMI-1640 media was dispensed across the surface of the well to dislodge any remaining spheroids, then media was collected and passed again over the strainer. This washing step was repeated three times prior to inverting the strainer, placing it over a well in a 6-well plate and rinsing with 4 ml of complete RPMI-1640 media. The spheroids were then collected individually using a P200 pipette and two spheroids were plated per well in a 12-well plate pre-coated with 200 μl of 8 mg/ml growth factor-reduced matrigel. The plated spheroids were then incubated at 37 °C with 5% CO2 for 48 h. Images were taken every 24 h under ×100 magnification using the Leica Inverted Light Microscope. Spheroid area and percent invasion (measured as the area of sprouts invading Matrigel) relative to the spheroid area was quantified using ImageJ software.

### 5-Ethynyl-2 deoxyuridine proliferation assay

Cellular proliferation was measured using EdU (5-ethynyl-2 deoxyuridine) incorporation. Briefly, glass coverslips, which had been sterilized *via* treatment with 70% ethanol solution for 15 min, were placed at the bottom of each well in a 12 well plate. Next, 50,000 cells suspended in 1 ml of RPMI- 1640 complete media were plated into each well and incubated overnight. The following day the medium was changed with fresh media containing 1 μl/ml EdU. The cells were then incubated at 37°C with 5% CO_2_ for 72 h and media was replaced with fresh EdU supplemented media every 24 h. After 3 days, cells were fixed using 4% paraformaldehyde solution and stained using Hoechst 33342 dye and the Click-iT EdU Cell Proliferation Kit for Alexa Fluor 488 dye (ThermoFisher Scientific, Cat No. C10337) according to manufacturer’s protocol. The cells were then imaged using fluorescence microscopy (Hoechst 33342 excitation/emission: 350/461 nm; Alexa Fluor 488 excitation/emission: 495/519 nm) and percent EdU-positive cells in each representative image were quantified using ImageJ software.

### Endothelial-like tube formation assay

Tube formation capabilities of HTR-8/SVneo cells were measured using an endothelial-like tube formation assay. Accelerated tube formation, conducted in the presence of VEGF-A is a measure of endovascular differentiation (Lala et al., 2012). Briefly, 20,000 HTR-8/SVneo cells were diluted in RPMI-1640 complete media supplemented with 30 ng/ml VEGF121 (Sigma, cat no. H9041), plated on 100 μl of 8 mg/ml Growth Factor Reduced Matrigel (Corning, Cat No. 354230) in a 24 well plate, and incubated at 37°C with 5% CO_2_ overnight. Cells were then imaged at 24 h under ×100 total magnification using the Leica Inverted Light Microscope. Tube length and branch points were quantified using ImageJ software.

### Statistical analysis

Statistical analyses were performed using GraphPad Prism Software version 8. Student’s *t*-test was used to measure differences between two means. All figures present mean data with error bars extending to ± the standard error of the mean (SEM). Significance is represented by: *p ≤ 0.05, **p ≤ 0.01, ***p ≤ 0.001, and ****p ≤ 0.0001.

## Results

### Decorin upregulated expression of microRNAs miR-512-3p

**FIGURE 1:**
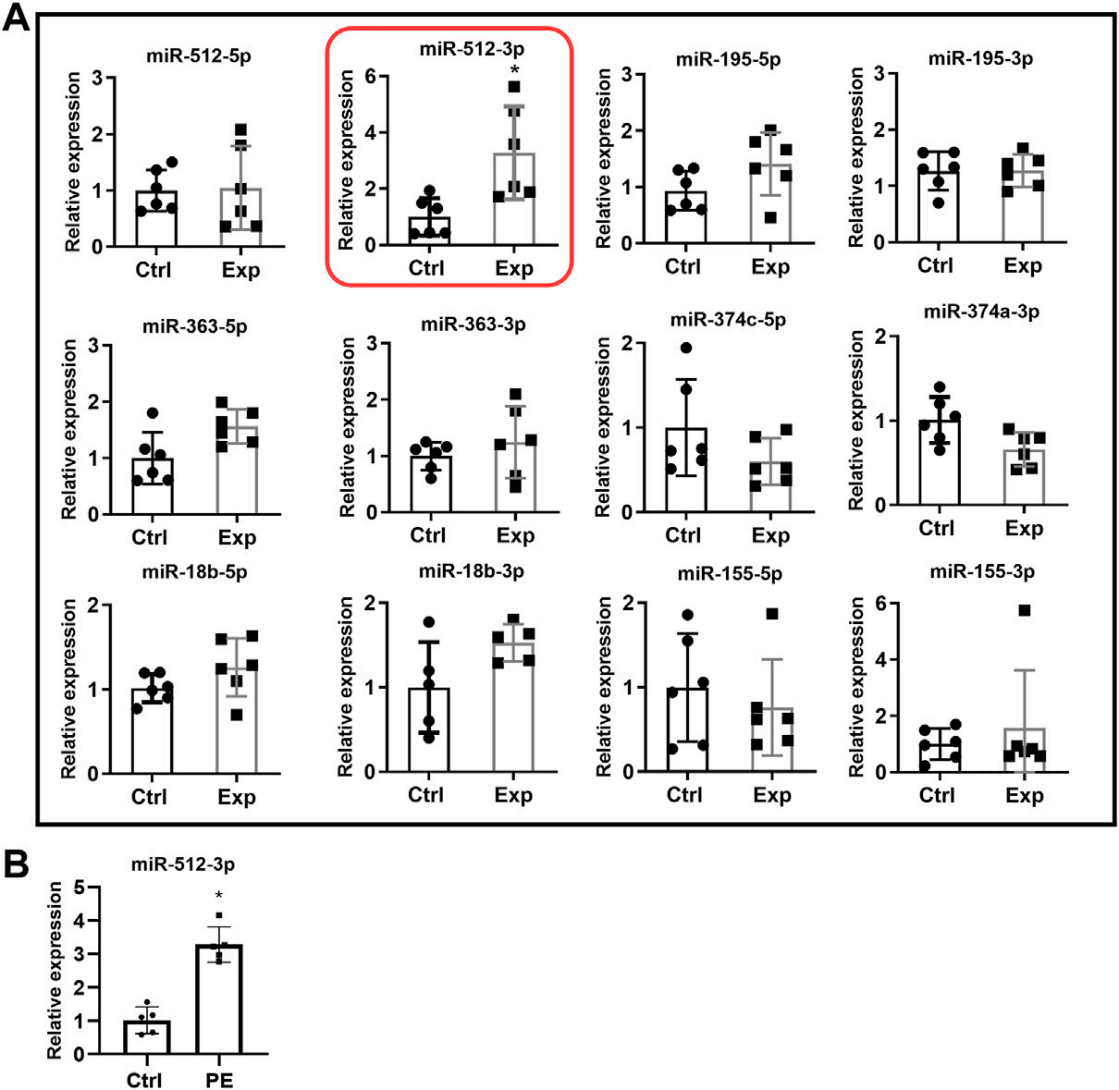
**(A)** MiRNA expression in HTR-8/SVneo cells in the presence or absence of exogenous DCN. HTR-8/SVneo cells were exposed to vehicle (Ctrl) or 250 nM DCN (Exp) for 24 h, and then qRT-PCR was used to measure expression of select miRNAs. Only miR-512-3p was significantly upregulated. Ctrl: cells not exposed to DCN. Exp: cells exposed to DCN. Time frame used for experiment: 24 h (N = 4–6, *p < 0.05). **(B)** Increased expression of miR-512-3p in PE placentas. Relative expression of miR-512-3p based on qRT-PCR analyses of placenta tissue samples derived from healthy normotensive control (Ctrl) and preeclamptic (PE) pregnancies. Analysis performed using Student’s t-test. Error bars extending to ± SEM. (*p ≤ 0.05, *n* = 5 biological replicates).

Trophoblast cells do not produce DCN. To make trophoblast cell exposed to DCN continuously, we generated DCN overexpressing HTR-8/SVneo cell line (WT-HTR-DCN), which steadily produces substantial amounts of DCN. Since we did not have an *a priori* knowledge of the local concentration of DCN produced by the decidua to which trophoblast is exposed, we reasoned that a sustained exposure to DCN resulting from ectopic introduction of DCN gene into the trophoblast would provide us with a good means of identifying the DCN-induced miRNAs. Next, we used a concentration of exogenous DCN (250 nM), based on the DCN concentration found in the supernatant of DCN over-expressing trophoblast confirmed by qPCR and ELISA (160–275 nM/24 h) and our earlier findings that at 250 nM, maximal migration inhibition was reached, to treat wild-type trophoblast cells. The latter approach narrowed down the number of DCN-induced miRNAs as presented in Figure 1A. We conducted a differential gene/miRNA micro-array analysis using WT-HTR-DCN and the control (mock- transfected) cell line. Of the large number of DCN-dysregulated miRNAs, we selected some which showed 1.5-fold upregulation or downregulation and were reported to be dysregulated in PE (Table 3). To determine whether exogenous DCN altered expression of these miRNAs, HTR-8/SVneo cells were treated with 250 nM DCN for 24 h, and expression of select miRNAs was evaluated using qRT-PCR. This concentration was based on a pilot experiment conducted by Nandi et al., 2018 showing a maximal inhibitory effect of DCN was observed between 200 nM and 300 nM. We found that only miR-512-3p, out of 12 miRNAs tested, was significantly upregulated (*p* < 0.05, Figure 1A).

**TABLE 3:** Selected miRNAs from our microarray data along with their literature reference.

MiRNA	Our microarray data	Literature references
miR-512	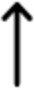	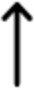 Wang et al. (2012)
miR-195	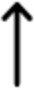	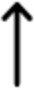 Chen and Wang (2013)
miR-18b	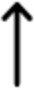	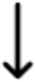 Wang et al. (2017)
miR-363	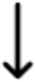	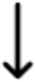 Zhang et al. (2015)
miR-374	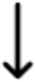	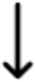 Li et al. (2013)
miR-155	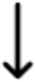	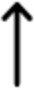 Gan et al. (2017)

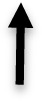
, indicates upregulation and 
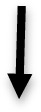
, indicates downregulation.

### miR-512-3p is overexpressed in preeclampsia placentas

To validate literature reports of increased miR-512-3p expression in PE, miRNA was extracted from gestational age matched placental tissues derived from healthy normotensive (control) pregnancies and PE complicated pregnancies. Purified miRNA was then used to synthesize cDNA for qRT-PCR analysis of miRNA expression level. miR-512-3p expression levels in PE placentas was found to be significantly higher than in control placentas (*p* ≤ 0.05, *n* = 5 biological replicates, Figure 1B).

### Overexpression of miR-512-3p resulted in inhibition of trophoblast migration, invasion, proliferation and endothelial like tube formation

**FIGURE 2:**
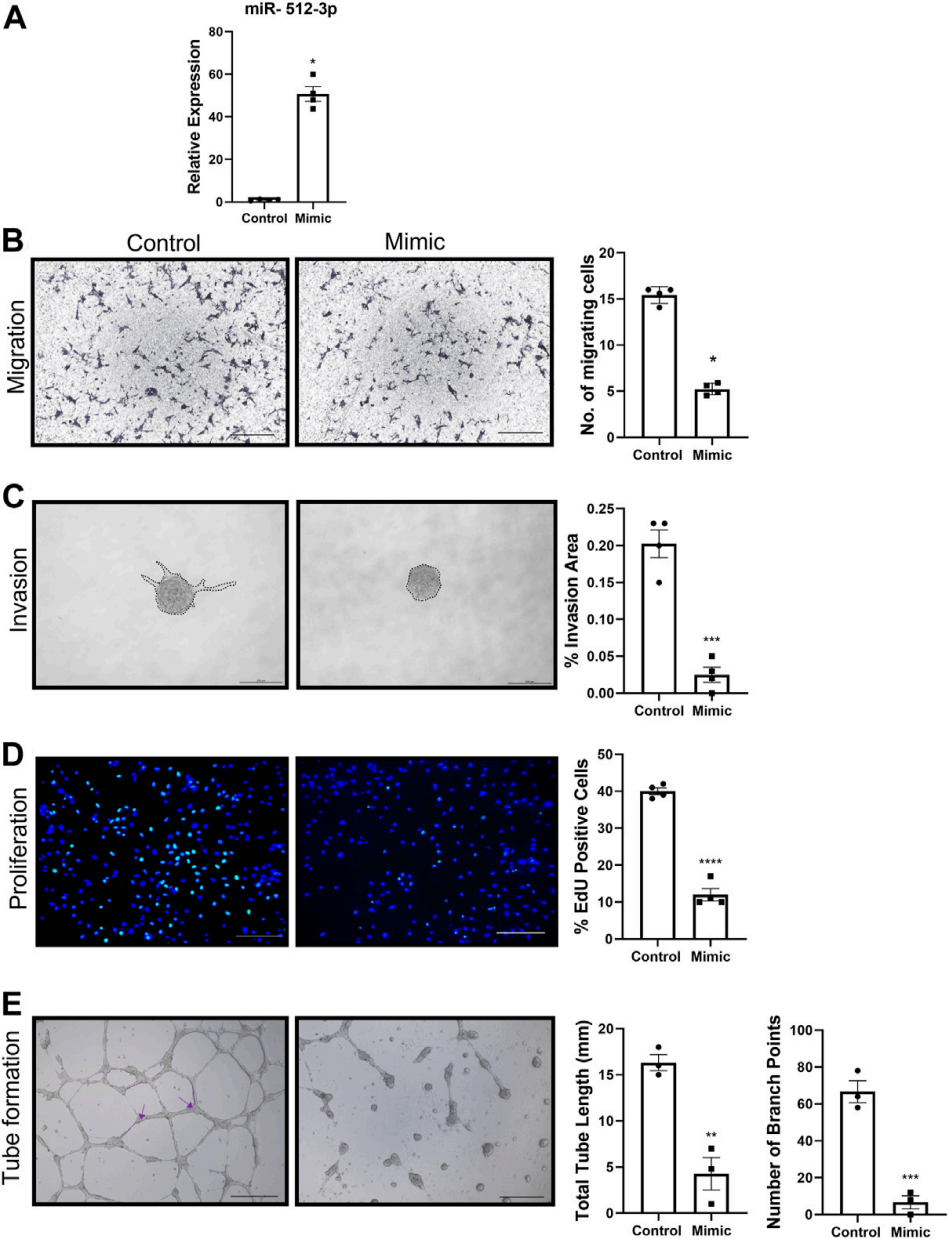
miR-512-3p overexpression repressed all HTR-8/SVneo trophoblast functions. **(A)** Expression of miR-512-3p in mock-transfected HTR-8/SVneo cells (Control) or following transfection with miR-512-3p (Mimic). (*p < 0.05, *n* = 3 replicates). **(B)** Migration measured using transwell assay. Representative images at 20× of membranes after 24 h with migrated cells stained dark purple (noted here as dark spots) for both control and miRNA overexpressing cells. The assay was quantified by calculating number of cells (×10^3^) migrating through the membrane post 24 h of starting the assay. Error bars extending to ± SEM. (*p ≤ 0.05, *n* = 4 replicates). **(C)** Invasion measured using spheroid invasion assays. Representative images of HTR-8/SVneo cells at ×100 total magnification (200 µm scale bar) following 24 h of growth on GFR matrigel. Invasion quantified by measuring percent invasion area (μm^2^) under control and mimic conditions at 24 h (***p ≤ 0.001, *n* = 3 replicates). **(D)** Proliferation measured using the 5-Ethynyl-2 Deoxyuridine (EdU) assay. Representative images of HTR-8/SVneo cells at ×100 total magnification (200 µm scale bar) following 72 h of EdU treatment. Nuclei stained with Hoechst (blue) and proliferating cells with incorporated EdU stained with Alexa Fluor™ 488 dye (green). Proliferation quantified by counting percent EdU-positive cells under control and mimic treated conditions. (****p ≤ 0.0001, *n* = 4 replicates). **(E)** Endovascular differentiation measured using endothelial-like tube formation assays. Representative images of HTR-8/SVneo cells at ×100 total magnification (200 µm scale bar) following 24 h of VEGF treatment and growth on GFR Matrigel. Tube formation quantified by measuring total tube length (mm) (shown by dotted line) and total number of branch points (shown by arrows) under control and mimic treated conditions. *p* ≤ 0.01, (***p ≤ 0.001, *n* = 3 replicates). All analysis performed using Student’s *t*-test. Error bars extending to ± SEM.

To investigate the role of miR-512-3p on various EVT functions, a miR-512-3p mimic was transfected into HTR-8/SVneo cells. Compared to mock-transfected cells, HTR-8/SVneo cells transfected with the miR-512-3p mimic had a 50-fold increased expression of miR-512-3p (Figure 2A, *p* ≤ 0.05, *n* = 4). Cells with increased miR-512-3p had reduced capacity to migrate through a transwell membrane compared to control cells (Figure 2B, *p* < 0.05, *n* = 4), showing that miR-512-3p has an inhibitory effect on trophoblast motility. To study the role of miR-512-3p on EVT invasion, a 3D spheroid invasion assay was done. Quantification of percent invasion (based on area of sprouts) relative to spheroid area revealed a significant decrease in the invasion capacity of HTR-8/SVneo cells over-expressing miR-512-3p at 24 h (Figure 2C, *p* ≤ 0.001, *n* = 4 replicates). Cells transfected with the miR-512-3p mimic also showed decreased numbers of proliferating EVT cells in comparison to the mock-transfected control cells, as indicated by reduced EdU fluorescence signals (Figure 2D, *p* ≤ 0.0001, *n* = 4 replicates). To investigate the role of miR-512-3p on EVT endovascular differentiation, an endothelial-like tube formation assay was performed. Extensive tube formation was observed under the control conditions; however, this was drastically disrupted in cells over-expressing miR-512-3p, including a significant decrease in both the total tube length and the number of branch points (Figure 2E, *p* ≤ 0.01, *n* = 3 replicates). Collectively, these results indicate that miR-512-3p compromised proliferative, migratory, invasive and tubulogenesis functions of EVT cells.

### Knockdown of miR-512-3p resulted in increase in proliferation

**FIGURE 3:**
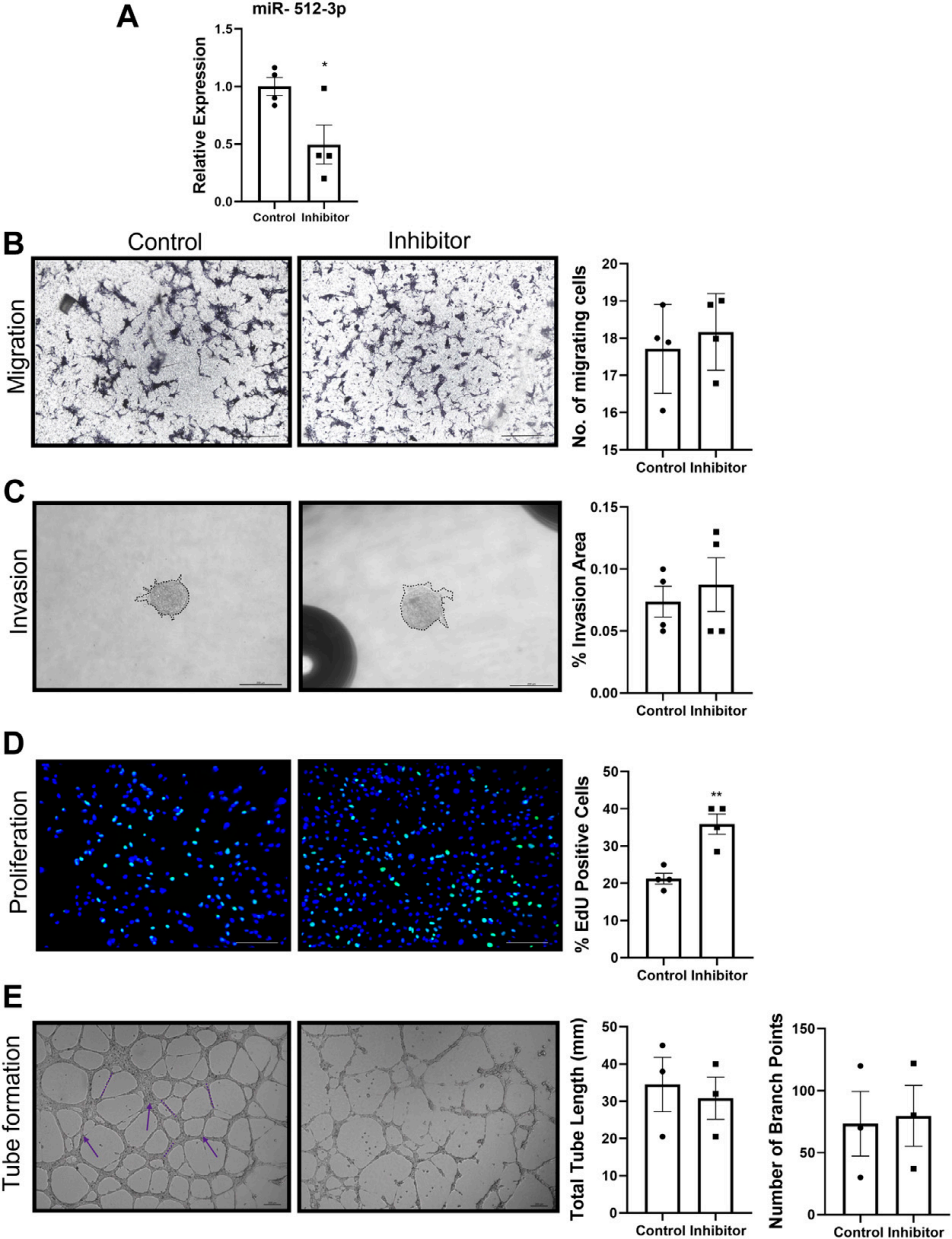
miR-512-3p knockdown promoted trophoblast proliferation. **(A)** Expression of miR-512-3p in mock-transfected HTR-8/SVneo cells (Control) or following transfection with miR-512-3p antisense oligonucleotide (Inhibitor). (*p* < 0.05, *n* = 3 replicates). **(B–E)** All assays were conducted and quantified as indicated in figure legend 2. Migration **(B)**, invasion **(C)** and Tube formation **(E)** did not show any significant difference between control and miR-512-3p inhibitor treated cells. (*n* = 3, 4) **(D)** Proliferation quantified by counting percent EdU-positive cells showed significant increase in inhibitor treated conditions vs. control. (**p ≤ 0.01, *n* = 4). All analysis were performed using Student’s *t*-test. Error bars extending to ± SEM.

Knockdown of miR-512-3p was performed by transfecting HTR-8/SVneo cells with a specific miR- 512-3p antisense oligonucleotide (inhibitor). Using this strategy, we achieved approximately 50% knockdown of miR-512-3p (Figure 3A, *p* < 0.05, *n* = 4). Migration, invasion, and tube formation did not show any significant change between the control and knockdown cells (Figures 3B,C,E, *n* = 3, 4). For the tube formation assay, there was some disruption in tube formation with the inhibitor, but the quantification as presented made no significant difference. However, cells with reduced miR-512-3p expression showed significant increase in proliferation as determined by the number of cells that incorporated EdU (Figure 3D, *p* < 0.01, *n* = 4). Collectively, these findings of an absence of significant changes in most EVT functions by miRNA knockdown may have resulted from the possibility that miR-512-3p is a DCN-inducible miRNA, expressed at relatively low levels in native EVT cells.

### Knocking down *PPP3R1*, a known target of miR-512-3p, increased migration and invasion of trophoblast cells

**FIGURE 4:**
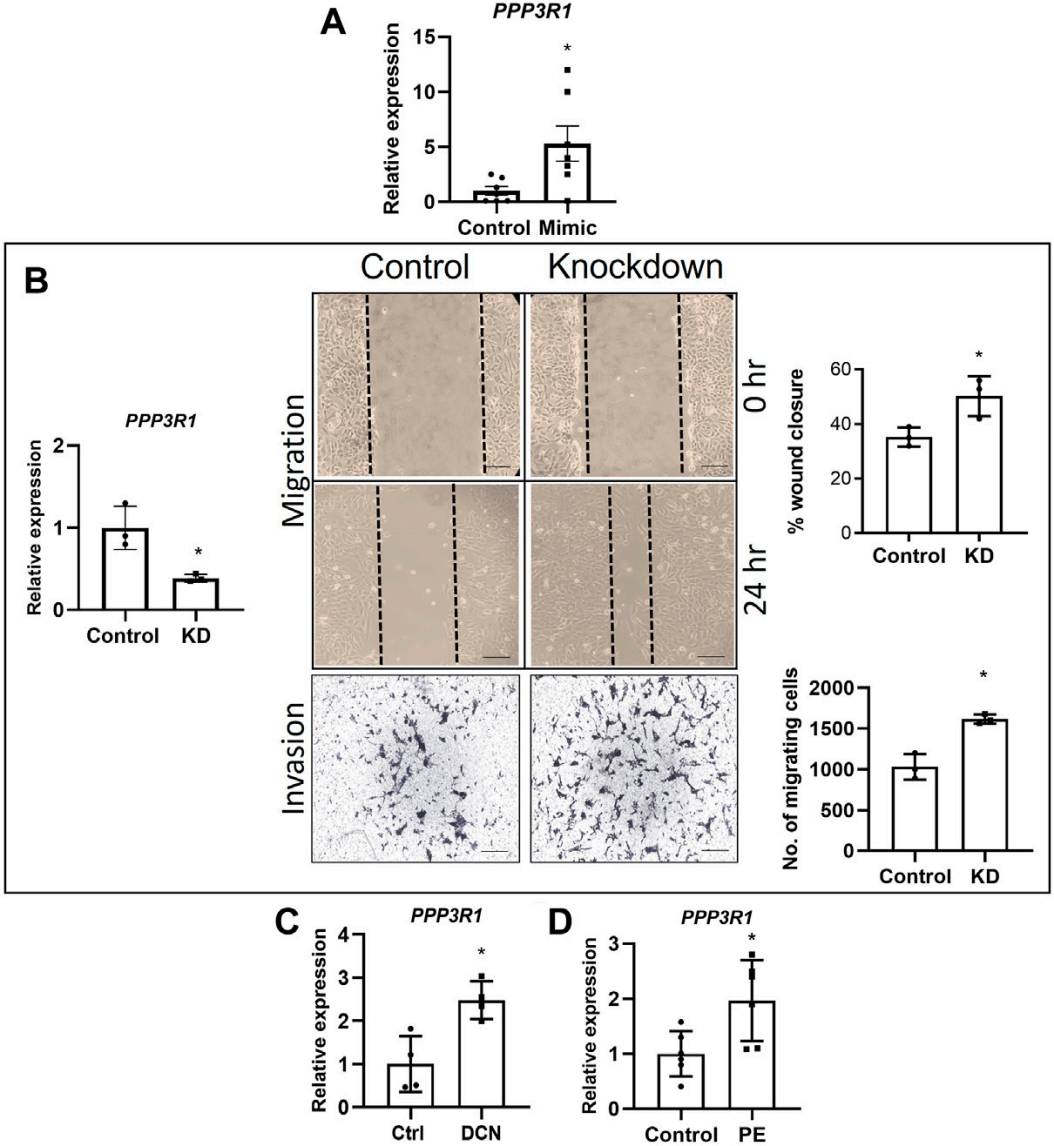
Roles of PPP3R1 in trophoblast functions. **(A)** miR-512-3p overexpression (miR-512-3p mimic) resulted in significant increase in *PPP3R1* confirmed by qRT-PCR (*p < 0.05, *n* = 7 replicates). **(B)** qRT-PCR showing knocking down *PPP3R1* in HTR-8/SVneo cells (60% knockdown, *p < 0.05) and the resulting effect on migration and Invasion assays. Migration assay performed using scratch assay. Images taken at 0 h and 24 h (20×), measured by the percentage of wound closure at 24 h showing significant increase in wound closure in *PPP3R1* knockdown cells. Invasion assay images (200 μm) shown for control and knockdown cells taken at 48 h measured by calculating the number of cells migrating through the Matrigel covered membrane. (*p < 0.05, *n* = 3). **(C)** qRT-PCR showing significant increase of *PPP3R1* mRNA expression after exogenous DCN treatment of HTR-8/SVneo cells (*p < 0.05, *n* = 4) and **(D)** in placentae from PE (*p < 0.05, *n* = 5). All analysis were performed using Student’s *t*-test. Error bars extending to ± SEM.

*PPP3R1* is a known target for miR-512-3p (Kurashina et al., 2014). The authors showed an downregulation in *PPP3R1* in miR-512-3p overexpressing BeWo choriocarcinoma cells. Paradoxically, our qRT-PCR data consistently showed an increase in *PPP3R1* mRNA in miR-512-3p overexpressing cells (Figure 4A, *p* < 0.05, *n* = 7). We proceeded with knocking down *PPP3R1* in HTR-8/SVneo cells (60% knockdown) and performed migration and invasion assays. There was a 50% increase in percent wound closure after 24 h in PPP3R1-knockdown cells in comparison to control cells (*p* < 0.05, *n* = 3). Invasion assay was performed using matrigel coated transwells at 48 h. There was a significant increase in the number of cells invading through the membrane in *PPP3R1*-knockdown cells in comparison to control (Figure 4B, *p* < 0.05, *n* = 3). Therefore, *PPP3R1* function in EVT cells was consistent with the upregulation of this gene by miR-512-3p overexpression. This finding called for investigating the possibility of an intermediary miR-512-3p binding molecule which targets *PPP3R1*.

### *PPP3R1* expression is upregulated in preeclampsia and decorin treated exposure of extravillous trophoblast cells

To the best of our knowledge, there is no information on the levels of *PPP3R1* in placentas from PE. Therefore, we decided to measure *PPP3R1* transcript levels using gestation age-matched control and PE placental tissues and found a significant increase of *PPP3R1* mRNA expression in PE-associated placentae (Figure 4C, *p* < 0.05, *n* = 5). Furthermore, since exposure of HTR-8/SVneo to DCN increased levels of miR-512-3p, and miR-512-3p overexpression increased *PPP3R1* expression, we also checked the expression level of *PPP3R1* after treatment with exogenous DCN for 24 h. We found a significant increase in *PPP3R1* mRNA after DCN treatment (Figure 4D, *p* < 0.05, *n* = 4).

### *PPP3R1* upregulation by miR-512-3p is intermediated by the transcription factor upstream transcription factor 2

**FIGURE 5:**
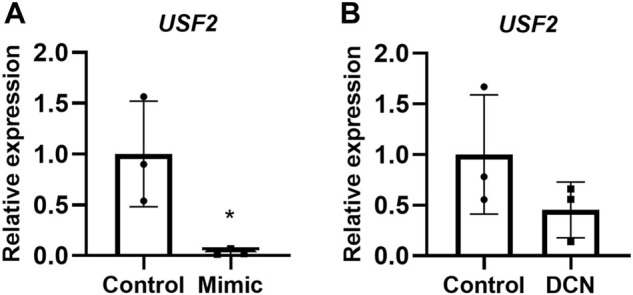
USF2 transcription factor may act as an intermediary for *PPP3R1* upregulation by miR-512-3p in HTR-8/SVneo trophoblast cells. qRT-PCR showing *USF2* upregulation in overexpressing miR-512-3p cells **(A)** and in exogenous DCN treated HTR-8/SV neo cells for 24 h **(B)** analyzed using Student’s t-test (*p < 0.05, *n* = 3).

Elevated expression of *PPP3R1* in miR-512-3p overexpressing cells indicated that this miRNA might be targeting negative regulators of *PPP3R1*. To identify these transcription factors, we used Enrichr, a tool that consists of both a validated user-submitted gene list and a search engine for transcription factors. By comparing miRNA target genes from TargetScan (https://www.targetscan.org/vert_80/), we identified *PPP3R1* regulatory transcription factors. Of these transcription factors, we identified USF2 as the only negatively regulated transcription factor targeting *PPP3R1*. *PPP3R1* was found to be a direct target of USF2. (https://maayanlab.cloud/Harmonizome/gene_set/USF2/ENCODE+Transcription+Factor+Targets) We validated this prediction by comparing *USF2* mRNA expression in control and miRNA-512-3p over-expressing trophoblast cells. There was a robust downregulation of *USF2* with a concomitant upregulation of *PPP3R1* in miR-512-3p over-expressing trophoblast cells (Figure 5A). Similarly exogenous DCN treatment (250 ng/ml for 24 h) tended to downregulate *USF2*, although this did not reach statistical significance (Figure 5B, *p* = 0.065).

## Discussion

PE is a life-threatening maternal pregnancy complication arising from abnormal placentation (Roberts and Lain, 2002). Poor placentation and uterine invasion by the trophoblast in PE severely reduce oxygen supply to the placenta and developing fetus, resulting in placental hypoxia and subsequent secretion of toxic factors into maternal circulation (Roberts and Lain, 2002). Recently, the proteoglycan DCN was shown to be overproduced by the decidua associated with PE and elevated in the plasma of PE patients during the second trimester predating PE (Siddiqui et al., 2016). DCN has been shown to facilitate development of placental hypoxia by impairing trophoblast proliferation, migration, invasion, and endovascular differentiation (Xu et al., 2002; Iacob et al., 2008; Khan et al., 2011; Lala et al., 2012). While these observations have been attributed to DCN interaction with several tyrosine kinase receptors EGFR, IGFR2, VEGFR2 (Iacob et al., 2008; Khan et al., 2011), the role of miRNAs in DCN actions on EVT cells remained unknown. With the observation that several miRNAs are dysregulated in PE pregnancies, the contribution of miRNAs to PE development has been gathering attention in recent years (Gao et al., 2018; Xu et al., 2021).

The current study aimed to identify DCN dysregulated miRNAs that could play a role in DCN mediated compromise in trophoblast functions associated with PE. We found multiple miRNAs that were altered in the miRNA microarray analysis with DCN-overexpressing trophoblast and selected those that were reported to be dysregulated in PE. Upon validation of selected miRNAs, we identified miR-512-3p that showed significant upregulation in DCN-treated native HTR-8/SVneo cells. We analyzed the expression of miR-512-3p miRNA in gestation age matched PE vs. control placental tissues (*n* = 5 for each). We found that this miRNA was significantly upregulated in placentas derived from PE pregnancies compared to normotensive controls. Although ours is a relatively small sample size, our findings are consistent with previous studies that reported upregulated expression of miR-512-3p in PE patients (Wang et al., 2012; Timofeeva et al., 2018).

The current study is first report on the roles of miR-512-3p on trophoblast functions such as migration, invasion, proliferation and tube formation (endovascular differentiation). We found that all these functions were variably downregulated by miR-512-3p. To ascertain that the transwell migration or invasion assay was not influenced by cell proliferation, we used mitomycin C treated cells in our assay. Similarly, to ascertain that spheroid invasion assay was not influenced by proliferation, we quantified the average spheroid area between 24 and 48 h for both control and over-expression conditions and did not find any significant change in spheroid area. Since Zou et al. (2015) reported that DCN can induce apoptosis, we ascertained that in all our functional assays the cell viability was 98%–99% as noted from trypan blue exclusion. We acknowledge that reduced cell viability is an indirect measure of apoptosis. Endothelial-like tube formation gave the same results both measured as total tube length and branching points. The effects of miR-512-3p on EVT functions have not been previously reported, other members of the C19MC cluster, such as miR-515-5p, have shown to may play inhibitory roles in trophoblast differentiation processes (Zhang et al., 2016). Knocking down miR-512-3p did not show any meaningful change in migration, invasion or tube formation. This may reflect relatively low expression of this miRNA in trophoblast cells in the absence of DCN. However, we observed a significant increase in trophoblast proliferation on miR-512-3p downregulated cells, indicating its dominant anti-proliferative role relative to other effects on the trophoblast.

We searched for potential targets of miR-512-3p using literature search and TargetScan. We narrowed down the targets by selecting those that are known to play a role in pregnancy or placenta development. We made a list of 18 targets and tried to validate them by qRT-PCR of control versus respective miRNA overexpressing cells. We validated a target (*PPP3R1*) for miR-512-3p. *PPP3R1* is a gene coding for calcineurin-B, and a known target of miR-512-3p (Kurashina et al., 2014). Calcineurin-B pathway has been reported to cause renal podocyte injury in PE (Yu et al., 2018).

Traditionally, miRNAs negatively regulate gene expression by repressing translation or directing sequence- specific degradation of target mRNAs (He and Hannon, 2004). But contrary to the traditional view, we consistently found an increase of *PPP3R1* in cells overexpressing miR-512-3p. Indeed, there is an increasing number of recent reports suggesting that miRNAs can also induce or promote the expression of target genes (Place et al., 2008; Rusk, 2008). For example, Huang et al. (2012) reported miR-744 and miR-1186 induced transcriptional activation by targeting promoter of Cyclin B1 gene. Another study by Xiao et al. (2017) showed that in HEK293T cell line, miR-24-1 overexpression increased histone 3 lysine 27 acetylation by targeting enhancers. Since *PPP3R1* was increased in HTR- 8/SVneo cells overexpressing miR-512-3p, the role of PPP3R1 on trophoblast functions was tested. We found a significant increase migration and invasion in *PPP3R1*-knockdown cells in comparison to control cells. This suggested that *PPP3R1* is an anti-migratory and anti-invasive molecule which could contribute to the anti-migratory actions observed in HTR-8/SVneo cells overexpressing miR-512-3p. Calcineurin acts as a crucial connection between calcium signaling and the phosphorylation states of numerous important substrates. These substrates include, but are not limited to, transcription factors, receptors and channels, proteins associated with mitochondria, and proteins associated with microtubules (reviewed by Creamer, 2020).

**FIGURE 6:**
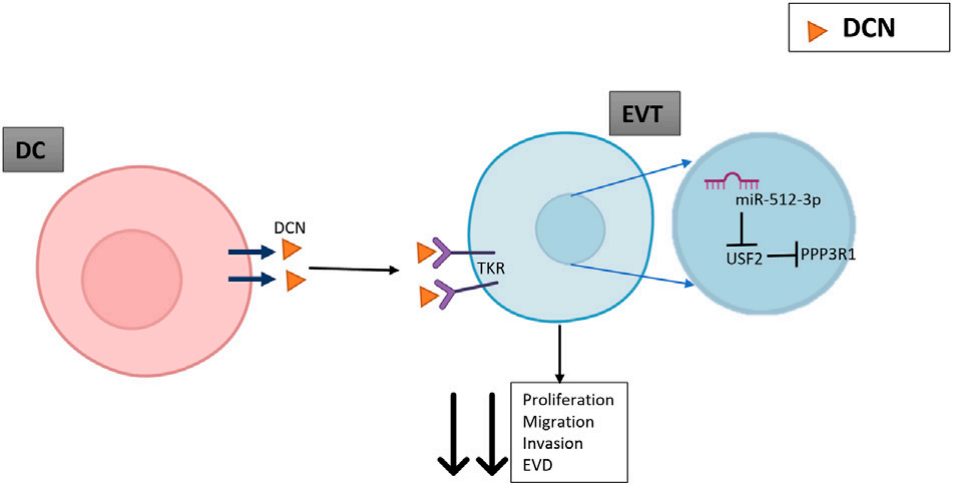
A schematic indicating the possible role of DCN mediated upregulation of *PPP3R1*. DCN released by decidual cells (DC) binds as a negative regulatory ligand to multiple tyrosine kinase receptors (TKR) on extravillous trophoblasts (EVT) to restrain migration, invasion, proliferation and endovascular differentiation (EVD). DCN also induces expression of miR-512-3p (binding partner unknown), which paradoxically upregulates its target *PPP3R1*. This is explained by its binding to an intermediary transcription factor USF2 which downregulates the expression of its target *PPP3R1* and results in decreased EVT proliferation, migration, and endovascular differentiation.

Using bioinformatics, we compared miR-512-3p target genes and *PPP3R1* regulatory transcription factors to search for potential intermediaries. The analysis revealed that miR-512-3p is a putative negative regulator of *USF2*, a transcription factor which represses expression of *PPP3R1*. Decreased expression of *USF2* and increased expression of *PPP3R1* in miR-512-3p overexpressing cells indicate that *PPP3R1* upregulation may result from an intermediary transcription factor *USF2*. It has been reported that DNA binding activity of *USF2* mediates the inhibitory effects of hypoxia on CYP19 gene expression to restrain cyotrophoblast differentiation into syncytiotrophoblast (Jiang and Mendelson, 2003). Present results reveal additional roles of *USF2* in regulating trophoblast functions. Whether PE- associated DCN over-expression is the cause of miR-512-3p upregulation in PE remains to be unequivocally established by an examination of the same subject population in a larger sample size. Taken together, our findings reveal a novel mechanism in miR-512-3p action (schema shown in Figure 6). While the binding partner for DCN-mediated miRNA induction in the trophoblast is currently unknown, it is also possible that DCN is a cargo carried by extracellular vesicles released from decidual cells (Ma et al., 2022) and endocytosed by EVT cells *in vivo.*

## Data Availability

The original contributions presented in the study are included in the article/Supplementary Material, further inquiries can be directed to the corresponding author.
